# Systematic review on the frequency of occurrence in nerve branches and the side of the face involved in trigeminal neuralgia and its clinical implication

**DOI:** 10.3389/fneur.2024.1362602

**Published:** 2024-03-27

**Authors:** Assefa Agegnehu Teshome, Gashaw Walle Ayehu, Berhanu Kindu Ashagrie, Nega Dagnaw Baye, Atalo Agemas Ayenew, Misganaw Asmamaw Mengstie, Taklo Simeneh Yazie, Asaye Alamneh Gebeyehu, Ermias Sisay Chanie, Yalew Melkamu Molla, Molla Taye Jemberie, Agmas Wassie Abate

**Affiliations:** ^1^Department of Biomedical Science, College of Health Sciences, Debre Tabor University, Debre Tabor, Ethiopia; ^2^Pharmacology and Toxicology Unit, Department of Pharmacy, College of Health Science, Debre Tabor University, Debre Tabor, Ethiopia; ^3^Department of Social and Public Health, College of Health Science, Debre Tabor University, Debre Tabor, Ethiopia; ^4^Department of Pediatrics and Child Health Nursing, College of Health Science, Debre Tabor University, Debre Tabor, Ethiopia; ^5^Department of Pediatrics and Child Health, College of Medicine and Health Science, University of Gondar, Gondar, Ethiopia; ^6^Department of Human Anatomy, College of Medicine and Health Science, University of Gondar, Gondar, Ethiopia; ^7^Department of Psychiatry, Dr. Ambachew Memorial Hospital, Amhara Regional Health Bureau, South Gondar Zone, Tach Gaynt, Ethiopia

**Keywords:** trigeminal neuralgia, trigeminal nerve branch, sidedness, fascial pain, systematic review

## Abstract

**Purpose:**

The purpose of this systematic review is to answer the focused question, “What is the commonly affected nerve branch and the side of the face involved in trigeminal neuralgia?”

**Types of studies reviewed:**

This systematic review included studies reporting commonly affected trigeminal nerve branches and the side of the face involved in trigeminal neuralgia. To find the potential studies published, the authors utilized specific search databases such as PubMed, and Google scholar.

**Results:**

Among 132 published studies, the authors selected only 11 to be included for this systematic review. The sample size ranged from 50 to 43,518 study subjects. This review identified that the mandibular branches of the trigeminal nerve were the most affected, followed by the maxillary branch. The review also identified that the right side of the face was predominantly affected.

**Conclusion and practical implications:**

The authors of this review identified a higher occurrence of trigeminal neuralgia in the mandibular division of the nerve, commonly affected on the right side of the face. Further prospective-based research and meta-analysis are required to validate the commonly occurring trigeminal nerve branch and sidedness of the face involved with its clinical implications in trigeminal neuralgia.

## Introduction

Trigeminal neuralgia (TN) can be defined as a neurological disorder characterized by sudden, unilateral, temporary, electric shock-like neuropathic facial pain in one or more distributions of the fifth cranial nerve. Although this definition was primarily based on expert opinions, TN can be classified into two forms: one that is “purely paroxysmal” and the other that is “with concomitant persistent facial pain” (1). There are also three types of TN: essential, idiopathic, and classical. Blood vessels compressing the trigeminal nerve root as it enters the brain stem is the cause of nearly all occurrences of trigeminal neuralgia (2). It has been demonstrated that not all patients with TN had vascular compression, and vice versa. Although neurovascular compression accounts for most TN cases, primary demyelination disorders, such as multiple sclerosis (MS), can also lead to TN symptoms (2–4). It is often known that women have trigeminal neuralgia at higher rates than men do (5). It has been well documented that patients with MS are at a higher risk of developing neuropathic pain and are estimated to be 20 times more likely to develop TN than the general population (6).

Due to the potential severity of the pain, debilitating effects of TN on the social life of the depressed patient, either physically, mentally, or socioeconomically, have been well documented in numerous studies (7, 8). TN can cause episodes of severe pain up to 50 times per day, which typically last for only a few seconds to 2 min (9). These TN episodes, which refer to the duration of repeated attacks, can vary from days to months, but they can also have lengthy remission intervals. The condition most commonly affects people over the age of 50 years, and women are more frequently affected than men (10). Non-neurologists such as dentists, general practitioners, internists, anesthesiologists, and neurosurgeons often treat individuals with trigeminal neuralgia. The patient's medical history and pain characteristics are the only factors used in the diagnosis. Due to a vast range of clinical symptomatology, delays or incorrect diagnoses frequently occur (11).

The symptoms of TN can be exacerbated by daily activities such as touching the skin lightly, washing, shaving, tooth brushing, blowing the nose, drinking hot or cold beverages, encountering a light breeze, applying makeup, smiling, and talking (12). The pathophysiology of TN is still mostly unclear. The current consensus on the underlying etiology of TN, also known as classical TN, is that it is caused by localized demyelination of the trigeminal nerve root entrance zone induced by compression from an aberrant artery or vein. Furthermore, it has been proven that TN is more common in patients with multiple sclerosis, leading to bilateral symptomatic or secondary TN (STN). Few published studies support a possible inheritance pattern of trigeminal neuralgia, implying that genetics may play a role in TN pathophysiology (13, 14).

Most previous studies revealed that the mandibular division was most commonly engaged, while the ophthalmic division was less frequently involved. The maxillary division persisted between the mandibular and ophthalmic divisions (15, 16). Some researchers have reported that atypical TN causes pain in the second and third divisions of the trigeminal nerve (16, 17). However, research ought to focus on pinpointing the precise causes of the affected side and the involvement of nerve branches. Previous literature reviews in this area are limited to specific involvement of the trigeminal nerve branch and the side of the face involved. Therefore, investigation of the affected nerve branches in trigeminal neuralgia might have paramount importance for early diagnosis and appropriate management. The aim of this systematic review was to answer the focused question, “What is the commonly affected nerve branch and the side of the face involved in trigeminal neuralgia?”

## Methods and materials

### Search strategy and study inclusion criteria

The searches were carried out by three independent authors (AA, ND, and BK) using the PubMed electronic database and Google scholar. In PubMed, we used the advanced search option to select relevant Medical Subject Headings (MeSH) terms. To obtain many results, we used the explode function, included all subheadings, and conducted a keyword search for each term using the multi-purpose (mp) function. Boolean operators combined the search terms. The results were limited to English language articles and studies conducted on humans (**Table 2**). AA and MA visually screened the titles and abstracts of the search results for eligibility according to the predefined criteria. The full text of the selected articles was then assessed to confirm eligibility. Articles that did not fulfill the criteria were excluded. In cases of ambiguous ideas, eligibility for inclusion was determined after a full-text review and discussion with the third party (YM, MT).

The terms ((“trigeminal neuralgia”[MeSH Terms] OR (“trigeminal”[All Fields] AND “neuralgia”[All Fields]) OR “trigeminal neuralgia”[All Fields]) AND (((“trigeminal nerve”[MeSH Terms] OR (“trigeminal”[All Fields] AND “nerve”[All Fields]) OR “trigeminal nerve”[All Fields]) AND (“branch”[All Fields] OR “branch s”[All Fields] OR “branche”[All Fields] OR “branched”[All Fields] OR “branches”[All Fields] OR “branching”[All Fields] OR “branching”[All Fields] OR “branches”[All Fields])) OR (“trigeminal nerve”[MeSH Terms] OR (“trigeminal”[All Fields] AND “nerve”[All Fields]) OR “trigeminal nerve”[All Fields] OR (“fifth”[All Fields] AND “cranial”[All Fields] AND “nerve”[All Fields]) OR “fifth cranial nerve”[All Fields]) OR (“5th”[All Fields] AND (“cranial nerves”[MeSH Terms] OR (“cranial”[All Fields] AND “nerves”[All Fields]) OR “cranial nerves”[All Fields] OR (“cranial”[All Fields] AND “nerve”[All Fields]) OR “cranial nerve“[All Fields])))) AND (fft[Filter]) were used in various combinations as primary search keywords.

Studies that provided information about the distribution of pain, the side of the face involved, clear descriptions regarding the diagnosis modalities, and the clearly identified trigeminal nerve branch involved in TN were included. However, studies that did not provide any specific report, consensus documents, or articles published before 2000 G.C. were excluded.

### Data extraction

After removing duplicates, title and abstract screening was conducted by three authors independently. After applying the selection criteria, all eligible studies with full texts were read, and the relevant references were checked manually. Three researchers independently extracted the data according to the inclusion and exclusion criteria, and the extracted data include the author's name, year of study, country, sample size, study design, frequency of the involved nerve branch, and the side of the face involved. When there was a difference of opinion, discussions with another author (GW) occurred until an agreement was reached. The extra reviewer confirmed the extracted data for accuracy.

### Quality and risk-of-bias assessment

The modified version of a quality assessment tool validated in a previous study was used to assess the qualities of the included studies. Three reviewers (AT, MM, and AWA) independently assessed the quality of the included studies. This review addressed the key domains of trigeminal neuralgia, including the side of the face involved, the affected trigeminal nerve branch, and the number of patients investigated. The discrepancies between three reviewers were resolved through discussion and there are articles that were included after consensus. The quality of the studies was assessed using the Newcastle-Ottawa Quality Assessment Scale (NOS). Based on the results of the quality assessment tool, the maximum score obtained from all questions was considered as having a low risk of bias. Total scores of 0–3, 4–6, and 7–9 were classified as low, moderate, and high risk of bias, respectively (28) (Table 1).

**Table 1:** Basic characteristics of the included studies.

**S.No**	**Author**	**Country**	**Study design**	**Sample size**	**Diagnostic criteria**	**Type of TN analyzed**	**NOS score**
1	Ayele et al. (18)	Ethiopia	Cross-sectional	61	ICHD	All types of TN	8
2	Jainkittivong et al. (19)	Thailand	Retrospective	188	ICHD	All types of TN	8
3	Yadav et al. (20)	India	Retrospective	72	Clinical and radiological	TITN	8
4	Thomas (21)	India	Retrospective	60	Hx, C/E and radiological	All types of TN	8
5	Debta et al. (22)	India	Retrospective	216	Clinical and radiological	All types of TN	8
6	Bölük et al. (23)	Turkey	Cross-sectional	9406	ICHD	All types of TN	7
7	El-Tallawy et al. (24)	Egypt	Cross-sectional	33285	ICHD	All types of TN	8
8	Shah et al. (16)	Pakistan	Cross-sectional	50	Hx, C/E, pain response to carbamazepine	All types of TN	7
9	Bangash (25)	Pakistan	Cross-sectional	100	Hx, C/E, pain response to carbamazepine	All types of TN	7
10	Maharjan et al. (26)	Nepal	Retrospective	80	ICHD	All type of TN	8
11	Maarbjerg et al. (27)	Denmark	Prospective	158	ICHD	CTN	8

ICHD, the international classification of headache disorders; Hx, history; C/E, clinical examination; TN, trigeminal neuralgia; CTN, classical trigeminal neuralgia.

### Statistical analysis

It was impossible to perform an appropriate meta-analysis because of the lack of research data among the studies related to this subject.

## Results

### Study selection and identification

**Figure 1:**
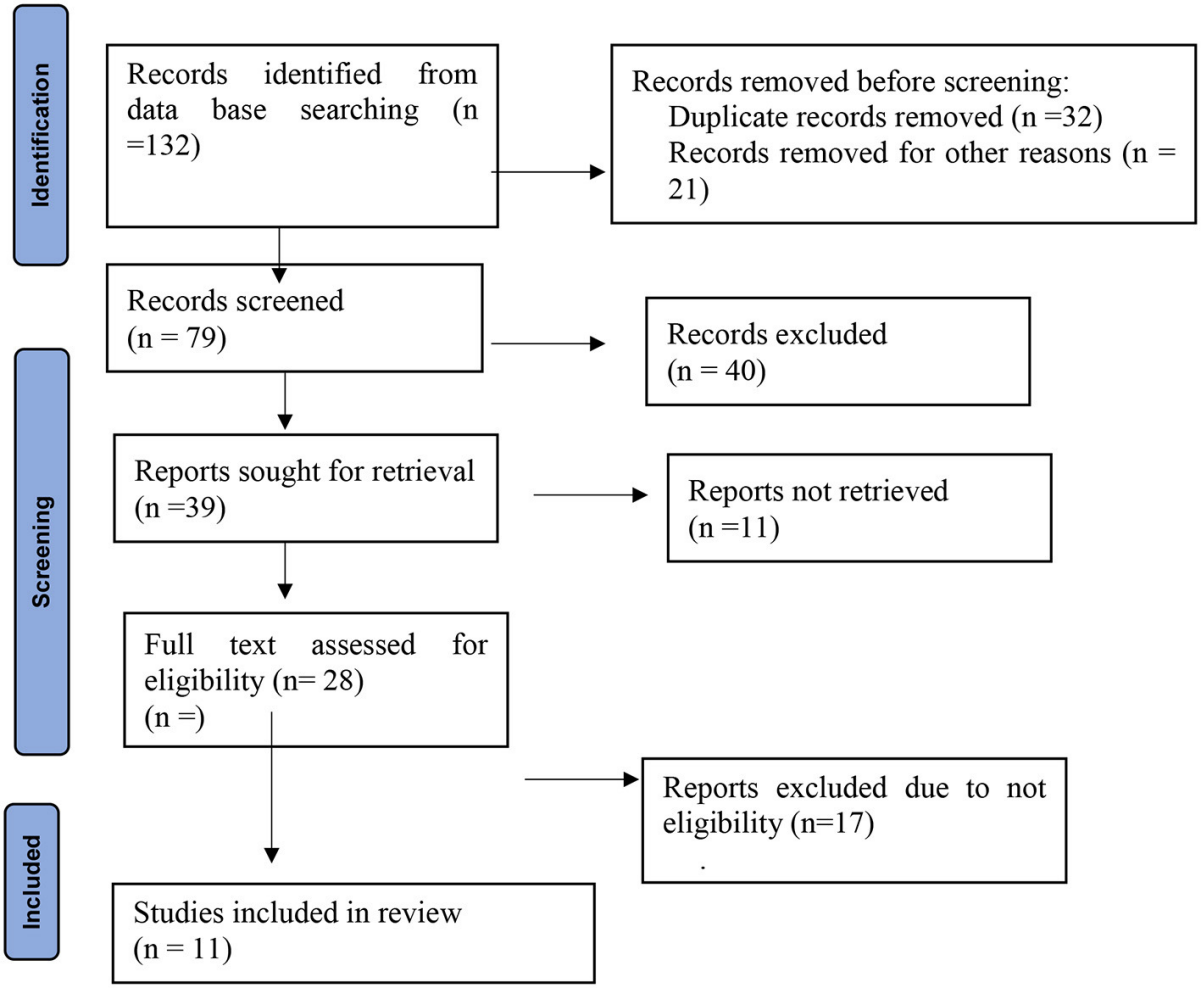
PRISMA flow diagram of article selection for systematic review on the frequency of occurrence in different nerve branches and the side of the face involved in trigeminal neuralgia.

Publications addressing the examination of the frequency of occurrence in different nerve branches and the side of the face involved in trigeminal neuralgia are included in this systematic review. A search of the database yielded 132 items in all. After the removal of articles due to duplications and other reasons, only 79 articles remained. After reading the abstract and the title, additional screening was conducted, and 40 articles were eliminated. Due to a lack of eligibility and non-retrieval of reports, 28 surviving items were again eliminated. As seen in the PRISMA flow diagram, 11 possible articles were ultimately included for qualitative synthesis (Figure 1).

### Characteristics of the included studies

Three of the 10 included studies were conducted in India (20–22), two studies were from Pakistan (16, 25), and one study each was conducted in Ethiopia (18), Turkey (23), Nepal (26), Egypt (24), Denmark (27), and Thailand (19). All of the included studies analyzed all types of TN, except for one study conducted in India that specifically analyzed idiopathic typical trigeminal neuralgia (ITTN) (20). A total of 43,676 study participants were included in the review. The studies contain a minimum and a maximum sample size of 50 and 33,285 participants, respectively. Regarding the diagnosis method of TN, all of the included studies relied on history, clinical examination, and radiological imaging as per the International Classification of Headache Disorders, 3rd edition, from the International Headache Society (ICHD-3/IHS) (10) (Table 1).

### Occurrence of trigeminal nerve branches and the side of the face involved

According to a study conducted in Ethiopia, it was found that TN affected the left side of the face in 29.5% of patients, the right side in 68.9% of patients, and both sides of the face in 1.6% of patients. The maxillary and mandibular branches (V2 + V3) were affected in 34.4% of the patients, while the mandibular branch (V3) was affected in half of the patients. No patient showed any evidence of a single ophthalmic branch involvement (V1) (18). Another retrospective study in Thailand showed that the mandibular division of the trigeminal nerve was shown to be the most commonly affected (30.3%) division, followed by the maxillary division alone (25%) and the combined maxillary and mandibular divisions (29.3%) (19).

The most commonly implicated branch was the mandibular division. Neuralgic discomfort limited to the mandibular distribution of the face was experienced by 56.9% of respondents. Of the patients who reported mandibular division involvement, 38.89% indicated involvement of the inferior alveolar nerve, and 18.05% experienced neuralgic pain limited to the mental nerve's distribution. Furthermore, 37.5% of patients experienced neuralgic pain in the maxillary nerve distribution, with all cases including the infraorbital nerve involvement, while 5.6% of patients experienced involvement of both the infraorbital and maxillary divisions on the same side as involvement of the mental and mandibular nerves (20).

Moreover, another study in Nepal assessed 100 TN patients and found that the right side of the face was found to be involved in 64% of them, while 36% of them had involvement on the left side. No case presented with bilateral involvement. The mandibular division was most commonly involved, accounting for 55%, followed by the maxillary (39%) and ophthalmic divisions (6%). The combination of V2 and V3 was seen in only 9% of patients. The combined involvement of all three divisions was not seen in this study (25).

Furthermore, a study conducted in Turkey using a large sample size found that 60% of cases had right-side involvement and 40% had left-side TN. It was also found that 20% of the patients had isolated maxillary involvement, 20% had isolated mandibular involvement, and 60% had combined maxillary and mandibular nerve involvement (23). Except for one study conducted in India (21) that found 60% of TN patients were affected by the maxillary nerve division, all other included studies reported that the mandibular division was the most commonly involved nerve, and the right side of the face was predominantly affected in all included studies (Table 2).

**Table 2:** Summary of different studies showing the frequency of occurrence in different nerve branches and the side of the face involved in trigeminal neuralgia.

**Author**	**Country**	**Major findings**
Ayele et al. (18)	Ethiopia	The patient's right side of the face was affected in 68.9% of cases, while the left side was afflicted in 29.5% of cases. Merely 1.6% of patients showed signs of bilateral facial involvement. Of the patients, 47.5% had involvement of the mandibular branch (V3), and 34.4% had involvement of both the maxillary and mandibular branches (V2 + V3). Not a single patient showed any evidence of an isolated ophthalmic branch (V1).
Jainkittivong et al. (19)	Thailand	More often than not, pain was felt on the right side of the face (1.8:1). The most commonly damaged division of the trigeminal nerve was the mandibular division (30.3%), which was followed by the maxillary division alone (25%) and the combined maxillary and mandibular divisions (29.3%).
Yadav et al. (20)	India	The branch that was most frequently implicated (56.9%) was the mandibular division. In the maxillary nerve distribution, neuralgic pain was reported by 37.5% of patients. On the same side, 5.6% of patients experienced problems with the maxillary and mandibular divisions. Moreover, 62.5% of patients had involvement on the right side, whereas only 37.5% of patients had involvement on the left side.
Thomas (21)	India	The most frequently affected branch was the maxillary nerve division distribution (41.7%), followed by the mandibular division (31.7%). The dual division (V2 + V3) was involved in 26.6% of patients. Right-side involvement was detected in 60% of patients, while left-side involvement was detected in 40% of patients.
Debta et al. (22)	India	The branch most often affected was the V3 nerve division distribution, accounting for 61.57%. Left-side involvement was detected in 45.83% of patients.
Bölük et al. (23)	Turkey	60% of TN patients had right-side involvement, while 40% of patients had left-side involvement. In addition, 20% of patients had only maxillary or mandibular involvement, while 60% of patients presented with both maxillary and mandibular nerve involvement.
El-Tallawy et al. (24)	Egypt	More patients had problems with their right side of the face than their left. In this investigation, the mandibular division was most frequently implicated, followed by the maxillary division.
Bangash (25)	Pakistan	It was shown that 36% of patients had involvement on the left side of the face and 64% had involvement on their right side of the face. There was not a single case that involved bilaterally. In this study, the mandibular division was most frequently engaged (55%), while the ophthalmic division was least frequently involved (6%).
Maharjan et al. (26)	Nepal	The common side of face involvement was right (57.5%) followed by left (35%), bilateral (5%), and anterior regions (2.5%). The most common trigeminal branch involved was the mandibular branch (42.5%) followed by the maxillary branch (41.25%). Furthermore, 16.25% of patients were affected by in combination of the two divisions.
Shah et al. (16)	Pakistan	In 64% of patients, the right side of their face was involved, while in 36% of them, the left side was involved. The most frequently affected division was the mandibular division (60%) followed by the maxillary (34%) and the ophthalmic (6%) divisions. In merely 8% of patients, V2 and V3 were present together. Not all the three divisions were involved in combination.
Maarbjerg et al. (27)	Denmark	In 56% of TN patients, the right side of their face was involved, while in 41% of them, the left side was involved. It affected solely the V2 and/or V3 trigeminal branches in 69% of TN patients, while the first branch alone was affected in only 4%. Notably, 49% had concomitant persistent pain in addition to paroxysmal stabbing pain.

TN, trigeminal neuralgia; V1, ophthalmic; V2, maxillary; V3, mandibular.

## Discussion

Although it is uncommon, TN lowers a person's quality of life when it does occur. Facial pain is the main TN symptom, which can make daily tasks such as eating and drinking difficult. The type and frequency may negatively influence a patient's quality of life and intensity of their pain, and pain may be linked to depression (11, 29, 30).

Our systematic review investigated the available evidence on the frequency of occurrence in trigeminal nerve branches, diagnosis modalities, and the sidedness of the face involved in trigeminal neuralgia. A lack of evidence about the commonest branches of the trigeminal nerve and the sidedness of the face involved in TN were confirmed as we reviewed the literature.

Furthermore, our determination of the frequency of occurrence in nerve branches, diagnosis modalities, and the sidedness of the face involved was hindered due to the complexity of identification and the variability of disease characteristics. In addition, we discovered that, due to these complications, it was not possible to have a comprehensive understanding of the phenomenon in a few studies that were restricted to reporting some variables rather than quantifying the occurrence of common nerve branches and the sidedness of the face involved.

Based on the reviewed literature, TN is diagnosed based on clinical grounds; therefore, a thorough clinical history, with special attention to the patient's description of symptoms, is the first essential step in the diagnosis process (31). The majority of research studies that have been published indicated that the ocular division (V1) was less frequently exhibited and that the mandibular division (V3) was most frequently implicated, followed by the maxillary division (10, 18, 19, 23, 32, 33). Furthermore, this systematic review revealed that the right side of the face is more frequently affected than the left side in 90% of the included articles, and this could be due to the fact that the foramen rotundum and foramen ovale on the right are narrower than those on the left (32).

This systematic review highlights the variability in the diagnosis modalities and summarizes the frequency of affected trigeminal nerve branches and involved facial sides that have been treated using medical and surgical interventions for TN by clinicians to date.

It is important to note that this review has a few shortcomings. Initially, very few studies fulfilled the strict requirements. Second, most of the reviewed studies were based on the retrospective study design. There is no meta-analysis in this review, which could offer stronger support than a systematic review alone. Therefore, more epidemiologic research with verified TN diagnostic standards and meta-analyses are necessary.

## Data availability statement

The original contributions presented in the study are included in the article/supplementary material, further inquiries can be directed to the corresponding author.

## Author contributions

AT: Writing – review & editing, Writing – original draft, Software, Methodology, Formal analysis, Conceptualization. GW: Writing – review & editing, Methodology, Formal analysis. BA: Writing – original draft, Supervision, Investigation. NB: Conceptualization, Methodology, Software, Writing – review & editing. AAA: Writing – original draft, Supervision, Investigation, Data curation. MM: Conceptualization, Methodology, Supervision, Writing – original draft. TY: Methodology, Supervision, Writing – original draft. AG: Writing – review & editing, Supervision, Methodology, Conceptualization. ES: Writing – review & editing, Methodology, Data curation, Conceptualization. YM: Supervision, Writing – review & editing, Conceptualization, Methodology. MT: Conceptualization, Supervision, Writing – review & editing. AWA: Writing – review & editing, Supervision, Methodology, Conceptualization.
